# Prospective observational study on Pazopanib in patients treated for advanced or metastatic renal cell carcinoma in countries in Asia Pacific, North Africa, and Middle East regions: PARACHUTE study

**DOI:** 10.1186/s12885-021-08738-z

**Published:** 2021-09-14

**Authors:** Erman Mustafa, Biswas Bivas, Danchaivijitr Pongwut, Chen Lingwu, Yoke Fui Wong, Tarek Hashem, Chun Sen Lim, Bulent Karabulut, Hsiao-Jen Chung, Chandra Chikatapu, Sara Ingles, Khemaies Slimane, Ravindran Kanesvaran

**Affiliations:** 1 Medical Oncology, Hacettepe University, Ankara, Turkey; 2 Medical Oncology, Tata Medical Center, Kolkata, West Bengal India; 3 Medical Oncology, Siriraj Hospital, Mahidol University, Bangkok, Thailand; 4 Medical Oncology, The First Affiliated Hospital of Sun Yat-sen, Guangzhou, Guangdong Province China; 5 Radiotherapy and Oncology, National Cancer Institute, Putrajaya, Malaysia; 6 Medical Oncology, Dr Tarek Hashem’s Clinic, Cairo, Egypt; 7 Clinical Oncology, Sultan Ismail Hospital, Johor Bahru, Malaysia; 8 Medical Oncology, Ege University, Izmir, Turkey; 9 Department of Urology, Taipei Veterans General Hospital and Department of Urology, College of Medicine and Shu-Tien Urological Research Cente, National Yang Ming Chiao Tung University, Taipei, Taiwan; 10 Oncology, Novartis Healthcare Pvt Ltd, Hyderabad, Telangana India; 11 Oncology, Novartis Pharma AG, Basel, Switzerland; 12 Oncology, Novartis Pharma SAS, Rueil-Malmaison, France; 13 Division of Medical Oncology, National Cancer Centre Singapore, Singapore, Singapore

**Keywords:** Renal cell carcinoma, Pazopanib, Real-world study, PARACHUTE

## Abstract

**Background:**

Clinical effectiveness and safety data of pazopanib in patients with advanced or mRCC in real-world setting from Asia Pacific, North Africa, and Middle East countries are lacking.

**Methods:**

PARACHUTE is a phase IV, prospective, non-interventional, observational study. Primary endpoint was the proportion of patients remaining progression free at 12 months. Secondary endpoints were ORR, PFS, safety and tolerability, and relative dose intensity (RDI).

**Results:**

Overall, 190 patients with a median age of 61 years (range: 22.0–96.0) were included. Most patients were Asian (70%), clear-cell type RCC was the most common (81%), with a favourable (9%), intermediate (47%), poor (10%), and unknown (34%) MSKCC risk score. At the end of the observational period, 78 patients completed the observational period and 112 discontinued the study; 60% of patients had the starting dose at 800 mg. Median RDI was 82%, with 52% of patients receiving < 85%. Of the 145 evaluable patients, 56 (39%) remained progression free at 12 months, and the median PFS was 10 months (95% CI: 8.48–11.83). 19% of patients (21/109) were long-term responders (on pazopanib for ≥18 months). The best response per RECIST 1.1 was CR/PR in 24%, stable disease in 44%, and PD in 31%. Most frequent (> 10%) TEAEs related to pazopanib included diarrhoea (30%), palmar-plantar erythrodysesthesia syndrome (15%), and hypertension (14%).

**Conclusions:**

Results of the PARACHUTE study support the use of pazopanib in patients with advanced or mRCC who are naive to VEGF-TKI therapy. The safety profile is consistent with that previously reported by pivotal and real-world evidence studies.

## Introduction

Pazopanib is an orally bioavailable adenosine triphosphate–competitive tyrosine kinase inhibitor (TKI) of vascular endothelial growth factor receptors (VEGFR) (1, 2, and 3), platelet-derived growth factor receptors (α and β), and c-Kit [1]. Pazopanib is approved by the Food and Drug Administration, European Medicines Agency, and other regulatory authorities as a monotherapy for patients with advanced renal cell carcinoma (RCC) and advanced soft tissue sarcomas [2, 3].

The efficacy of pazopanib has been demonstrated in two phase III randomised controlled trials [4, 5]. Pivotal phase III clinical trials are the best source of evidence for determining the efficacy of a treatment in a specific population; however, these studies cannot provide data for all possible clinical scenarios or disease characteristics. The real-world PRINCIPAL study demonstrated the effectiveness and safety of pazopanib in a routine clinical setting in patients with advanced or metastatic RCC [6]. Limited current knowledge on the use of pazopanib in routine clinical practice further highlights the need for real-world studies of pazopanib, especially for evaluating patients who have been under-represented in the registration trials.

PARACHUTE study aimed to describe the clinical effectiveness and safety of pazopanib in a real-world setting in patients with advanced or metastatic RCC who are naive to VEGFR-TKI therapy. This study was conducted across countries in Asia Pacific, North Africa, and Middle East regions, where data from registration trials were lacking or limited. The aim was to collect data from medical centres related to the management of patients treated with pazopanib for advanced or metastatic RCC.

## Patients and methods

PARACHUTE, a phase IV, prospective, non-interventional, observational study, did not impose a therapeutic protocol, diagnosis/therapeutic interventions, or a strict visit calendar. Patients diagnosed with advanced or metastatic RCC of any histology who were treated according to local practices and who were treated for the first time with pazopanib were included. Patients were treated with pazopanib in accordance with the summary of product characteristics [7]. The study consisted of an observational period from baseline to month 12. Patients were allowed to participate in other interventional clinical studies. All patients provided informed consent, and the study was conducted in accordance with the International Conference on Harmonisation Good Clinical Practice guidelines, patient privacy requirements, and ethical principles outlined in the Declaration of Helsinki 2013.

The study was designed to enrol approximately 180 patients with advanced or metastatic RCC who were naive to VEGFR-TKI therapy in 15 countries across Asia Pacific, Middle East, and North Africa. The sample size was based on the conservative assumption of survival, i.e., by taking the lower limit of the confidence interval (CI) of the PRINCIPAL and VEG105192 studies, along with increasing the margin of error to 7.7% [4, 6]. Assuming 15% of the data was incomplete or censored before month 12 owing to reasons other than disease progression, the minimum sample size required based on a conservative assumption of a progression-free survival (PFS) of 38% with a 7.7% of margin of error was 180 patients [4]. The enrolment was competitive, with 35 centres participating in this study.

Patients aged ≥18 years with advanced or metastatic RCC who were about to start new treatment with pazopanib on study entry or had already started new treatment with pazopanib within 15 days before study entry based on the treating physician’s decision were eligible for the study. Prior cytokine therapy was allowed, but prior anti-VEGF therapy for RCC was not allowed.

### Objectives and assessments

The primary objective of the study was to describe the clinical effectiveness of pazopanib in a real-world setting based on the proportion of patients remaining progression free at 12 months. Secondary objectives were to describe other clinical effectiveness factors for pazopanib: objective response rate (ORR) at 12 months, PFS, and response (ORR and progression-free response) based on the Memorial Sloan Kettering Cancer Center (MSKCC)/International Metastatic Renal Cell Carcinoma Database Consortium (IMDC) risk categories and age; gain information on treatment sequences; relative dose intensity (RDI) of pazopanib, collected in electronic case report forms; safety and tolerability of pazopanib; correlation of the RDI of pazopanib with its safety profile and treatment outcomes at 12 months. The exploratory objective of the study was to assess the proportion of patients who were long-term responders, i.e., those remaining progression-free for ≥18 months while receiving pazopanib treatment at the end of study (EOS).

The primary endpoint, i.e., the proportion of patients remaining progression-free at 12 months, was estimated along with the 95% CIs using the Kaplan-Meier method by means of the complementary log-log transformation [8, 9]. The Greenwood’s formula was used to calculate the standard error of the estimates from the Kaplan-Meier curve [8]. Secondary effectiveness variables were ORR, defined as the proportion of patients with a best response of complete response (CR) or partial response (PR) at 12 months for patients with measurable lesions evaluated using only the Response Evaluation Criteria in Solid Tumours (RECIST) version 1.1, and PFS, defined as the time from the date of the start of pazopanib therapy to the date of the event (defined as the first documented disease progression or death due to any cause) [10]. If a patient did not have an event, PFS was censored at the date of the last adequate tumour assessment. Long-term responders were defined as patients who were progression-free for ≥18 months while receiving pazopanib treatment. Secondary safety variables included safety and tolerability of pazopanib; RDI (defined as the ratio of the average daily dose of pazopanib to the recommended daily dose of pazopanib during the 12-month observational period); correlation of RDI with the safety profile; and treatment outcomes at 12 months.

Patients who received ≥1 dose of pazopanib (full analysis set [FAS]) were evaluable for efficacy, and all patients who received ≥1 dose of pazopanib and had ≥1 safety assessment on or after baseline (safety analysis set [SAS]) were evaluable for safety. The measurable disease population comprised patients with measurable disease at baseline and was used for the analysis of ORR.

### Statistical analysis

The proportion of patients remaining progression-free at 12 months was estimated along with the 95% CIs using the Clopper-Pearson method. The best overall response for overall and measurable disease was estimated using the sample proportion (%) along with the 95% CIs with the Clopper-Pearson method. The Kaplan-Meier method was used to analyse PFS. Adverse events (AEs) and concomitant medication terms were coded using the Medical Dictionary for Regulatory Activities (MedDRA).

## Results

A total of 200 patients from 15 countries were enrolled between June 2017 and December 2018. Ten patients were excluded owing to nonreliability of data, and 190 patients from 14 countries and 34 centres with a median (range) age of 61.0 (22.0–96.0) years were included in the final analysis. Most patients were Asian (70.0%), and clear cell carcinoma was the most common type of RCC (80.5%). Patient demographics are shown in Table 1.

**Table 1:** Patient demographics (SAS)

Demographic variable	Pazopanib ***N*** = 190
**Age, years**	
Mean (SD)	61.2 (12.3)
Median (range)	61.0 (22.0–96.0)
**Age category, n (%)**	
≥ 65 years	71 (37.4)
**Sex, n (%)**	
Male	133 (70.0)
**Race, n (%)**	
Caucasian	55 (28.9)
Black	2 (1.1)
Asian	133 (70.0)
**BMI, kg/m**^**2**^**,*n*** **= 120**	
Mean (SD)	24.8 (5.1)
Median (range)	23.9 (14.1–41.9)
**Disease status, n (%)**	
Metastatic	182 (95.8)
Locally advanced	8 (4.2)
**Predominant histology/cytology, n (%)**	
Clear cell carcinoma	153 (80.5)
Non-clear cell carcinoma	18 (9.5)
Papillary	12 (6.3)
Chromophobe	3 (1.6)
Invasive ductal carcinoma^a^	1 (0.5)
Unknown non-clear cell carcinoma^b^	2 (1.1)
Missing	2 (1.1)
Other^c^	17 (8.9)
**MSKCC prognostic, n (%)**	
Favourable	17 (8.9)
Intermediate	90 (47.4)
Poor	19 (10.0)
Unknown	64 (33.7)
**IMDC prognostic, n (%)**	
Favourable	15 (7.9)
Intermediate	80 (42.1)
Poor	22 (11.6)
Unknown	73 (38.4)
**Common (≥ 20%) site of metastases, n (%)**	
Lung	126 (66.3)
Lymph node	74 (38.9)
Bone	52 (27.4)
Liver	37 (19.5)
**Number of metastatic sites, n (%)**	
0	8 (4.2)
1	62 (32.6)
2	69 (36.3)
3	29 (15.3)
> 3	22 (11.6)
**Prior antineoplastic surgeries reported, n (%)**	**143 (75.3)**
**Prior antineoplastic medications reported, n (%)**	**14 (7.3)**
**Duration of prior antineoplastic medications, months, mean (SD)**	**3.7 (4.3)**

*BMI* body mass index, *IMDC* International Metastatic Renal Cell Carcinoma Database Consortium, *MSKCC* Memorial Sloan Kettering Cancer Center, *SAS* safety analysis set, *SD* standard deviation

Percentages are based on the total number of enrolled patients. BMI was calculated as body weight (in kilograms) divided by height (in square metres)

^a^metastatic disease

^b^Under histology, physician reported non-clear cell carcinoma for these 2 patients, no further classification available

^c^5 patients had details on histology including Clear and chormophobe cell type, Tfe3 translocation RCC, left kidney beilini collecting duct carcinoma, tubulocystic carcinoma and squamous cell carcinoma each (*n =* 1). It was reported unknown or unidentified in 12 patients

A total of 189 patients were included in the FAS and 190 in the SAS. Overall, 78 (41.1%) patients completed the observational period and 67 (35.3%) completed the follow-up period. The most common (≥ 5%) reasons for discontinuation were progressive disease (30.0%), death (13.2%), and AEs (5.3%). Patient disposition by the end of the observational period is presented in Table 2.

**Table 2:** Patient disposition at the end of observation (SAS)

Disposition/reason	Pazopanib ***N*** = 190 n (%)
**Enrolled**	190 (100.0)
**Completed observational period**	78 (41.1)
**Discontinued in observational period**	112 (58.9)
**Primary reason for discontinuation in observational period**	
Adverse event	10 (5.3)
Progressive disease	57 (30.0)
Lost to follow-up	8 (4.2)
Death	25 (13.2)
Patient/guardian decision	5 (2.6)
Physician decision	2 (1.1)
Withdrawal of consent	7 (3.7)
Unknown	5 (2.6)

*SAS* safety analysis set

End of observation was defined as the period from baseline to month 12; patients with multiple reasons for discontinuation were counted once for each reason; percentages were based on the total number of enrolled patients

### Efficacy

**Fig. 1:**
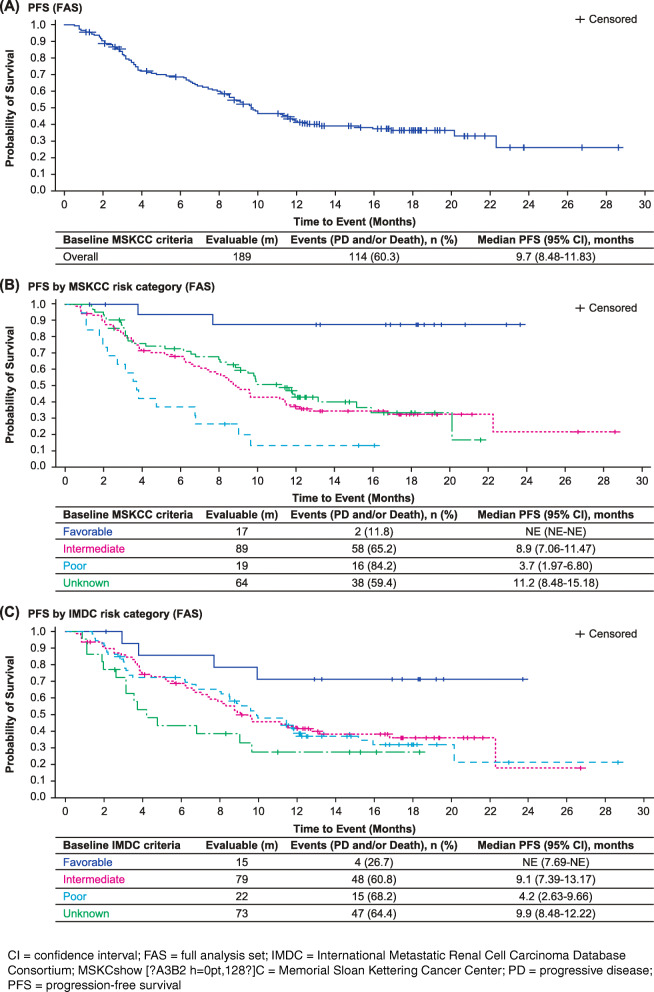
Kaplan-Meier plot for PFS for (**A**) all patients, (**B**) MSKCC risk categories, and (**C**) IMDC risk categories (FAS)

Of the 145 evaluable patients, 56 (39%; 95% CI: 30.7–47.2) remained progression-free at 12 months, and 37 (34%; 95% CI: 25.2–43.6) of the 109 evaluable patients remained progression-free at EOS. The overall median PFS was 9.7 months (95% CI: 8.5–11.8). The median time to progression was longer among patients aged < 65 years (11.2 months [95% CI: 9.5–15.9]) than among patients aged ≥65 years (6.8 months [95% CI: 4.2–9.9]). The median time to progression was longer for the intermediate categories (MSKCC: 8.9 months [95% CI: 7.1–11.5]; IMDC: 9.1 months [95% CI: 7.4–13.2]) than for the poor categories (MSKCC: 3.7 months [95% CI: 2.0–6.8]; IMDC: 4.2 months [95% CI: 2.6–9.7]). Median not reached in favourable group and median time to progression in unknown risk categories was comparable with the overall patients (MSKCC: 11.2 months [95% CI: 8.48–15.18]; IMDC: 9.9 months [95% CI: 8.48–12.22]) Fig. 1 shows PFS by baseline by MSKCC and IMDC criteria.

Among the 127 patients with available RECIST data, the most commonly reported best objective response by EOS was stable disease (56 patients [44.1%]), followed by progressive disease (PD; 40 patients [31.5%]), PR (27 patients [21.3%]), and CR (4 [3.1%] patients). The ORR (CR + PR) for the overall population was 24.4%. The disease control rate (DCR; CR + PR + stable disease) was 68.5%. For patients with measurable disease, the ORR was 25.7% and DCR was 67.3% (Table 3).

**Table 3:** Best response among patients with overall and measurable disease (FAS)

Disease	Overall response rate n (%)	Complete response n (%)	Partial response n (%)	Stable disease n (%)	Progressive disease n (%)	Total n (%)
**Overall**^**a**^**(*n*** **= 127)**						
n (%)	31 (24.4)	4 (3.1)	27 (21.3)	56 (44.1)	40 (31.5)	127 (100.0)
95% CI	17.2–32.8	0.9–7.9	14.5–29.4	35.3–53.2	23.5–40.3	97.1–100.0
**Measurable**^**b**^**(*n*** **= 113)**						
n (%)	29 (25.7)	3 (2.7)	26 (23.0)	47 (41.6)	37 (32.7)	113 (100.0)
95% CI	17.9–34.7	0.6–7.6	15.6–31.9	32.4–51.2	24.2–42.2	96.8–100.0

*FAS* full analysis set, *CI* confidence interval, Overall response rate = CR + PR

^a^Patients with or without measurable disease at baseline and response data available as per RECIST v1.1

^b^Patients with measurable disease at baseline as per RECIST v1.1 and response data available as per RECIST v1.1

Patients in the < 65 years age group were more likely to achieve CR or PR as best response (27.5%) than patients in the ≥65 years age group (19.1%). Among patients with MSKCC and IMDC risk data available at baseline, the highest proportion of patients who achieved CR or PR as best response was the favourable group (7/12; 58.3% in the MSKCC risk group and 50.0% (4/8) in the IMDC risk group) (Table 4).

**Table 4:** Summary of best response by age and prognostic group categories (FAS)

	Overall response rate n (%)	Complete response n (%)	Partial response n (%)	Stable disease n (%)	Progressive disease n (%)
**MSKCC risk categories (*n*** **= 93)**					
Favourable (*n =* 12)	7 (58.3)	1 (8.3)	6 (50.0)	5 (41.7)	0
Intermediate (*n* = 69)	18 (26.1)	2 (2.9)	16 (23.2)	30 (43.5)	21 (30.4)
Poor (*n =* 12)	0	0	0	5 (41.7)	7 (58.3)
**IMDC risk categories (*n =*** **80)**					
Favourable (*n =* 8)	4 (50.0)	0	4 (50.0)	4 (50.0)	0
Intermediate (*n* = 61)	16 (26.2)	2 (3.3)	14 (22.9)	26 (42.6)	19 (31.1)
Poor (*n =* 11)	1 (9.1)	0	1 (9.1)	4 (36.3)	6 (54.5)
**Age categories (*n =*** **127)**					
< 65 years (*n =* 80)	22 (27.5)	3 (3.8)	19 (23.7)	33 (41.3)	25 (31.2)
≥ 65 years (*n* = 47)	9 (19.1)	1 (2.1)	8 (17.0)	23 (48.9)	15 (31.9)

*FAS* full analysis set, *IMDC* International Metastatic Renal Cell Carcinoma Database Consortium, *MSKCC* Memorial Sloan Kettering Cancer Center, Overall response rate = CR + PR

### Treatment sequence profile

Overall, 14 (7.3%) patients had received prior treatment for RCC before switching to pazopanib. The most frequent prior treatment was interferon (12 of 15 patients [80.0%]); all other types of prior treatments were received by 1 patient (6.7%) each. Disease progression was the most common reason reported for switching to pazopanib (11 patients [73.3%]) (Table 5).

**Table 5:** Summary of profile treatment sequences before and after pazopanib (SAS)

	Pazopanib ***N =*** 190 n (%)
**Patients receiving treatments before pazopanib**	**14 (7.3)**
Interferon	12 (6.3)
Nivolumab and ipilimumab	1 (0.5)
Epacadostat	1 (0.5)
Pembrolizumab	1 (0.5)
**Patients receiving treatments after discontinuing pazopanib**	**52 (27.4)**
Nivolumab	21 (40.4)
Axitinib	13 (25.0)
Everolimus	13 (25.0)
Sunitinib	9 (17.3)
Cabozantinib	5 (9.6)
Lenvatinib	3 (5.8)
Sorafenib	2 (3.8)
**Reasons for switching to pazopanib**	**15 (7.9)**
Disease progression	11 (73.3)
Lack of tolerability	2 (13.3)
Other	1 (6.7)
Physician decision	1 (6.7)
**Reasons for switching from pazopanib**	**52 (27.4)**
Disease progression	45 (86.5)
Adverse event	7 (13.5)

*SAS* safety analysis set

A total of 52 patients (27.4%) received treatment after discontinuing pazopanib. The most common (≥10.0%) treatments were nivolumab (21 patients [40.4%]), axitinib (13 patients [25.0%]), everolimus (13 patients [25.0%]), and sunitinib (9 patients [17.3%]). Disease progression was the most common reason reported for switching from pazopanib (45 patients [86.5%]) followed by AEs (7 patients [13.5%]) (Table 5).

### Exposure to pazopanib

The starting dose of pazopanib was 800 mg (full dose) in 63.2% of patients, 600 mg in 22.1%, and 400 mg in 14.7%. Overall, 5.8% of the patients receiving 400 mg/day and 3.2% receiving 600 mg/day of pazopanib had a dose increase, while 10.5% of patients receiving 800 mg/day and 2.1% receiving 600 mg/day had a dose decrease. From the initial prescription of pazopanib, the overall mean (standard deviation [SD]) exposure to pazopanib was 10.1 (6.79) months.

The mean (SD) overall RDI was 79.4% (21.04), which was close to the median RDI of 81.9%. The minimum RDI was 32.5% and maximum was 100%. A total of 120 patients (63.2%) had a reduced RDI (< 100%), of whom 99 (52.1%) had an RDI of < 85%. A higher proportion of patients achieving CR or PR (32.7%) and a lower proportion of patients with PD (23.6%) were seen with a mean RDI of ≥85% vs a mean RDI of < 85% (ORR: 19.2%; PD: 37.0%). In patients who attained CR or PR, the overall mean (SD) RDI was 83.1% (19.4). The mean (SD) RDI in patients with an RDI of < 85% was 63.7% (12.8), and in patients with an RDI of ≥85%, the mean (SD) RDI was 98.2% (3.4). Patients with a mean RDI of ≥85% were less likely to experience an AE (76.9%) or a serious AE (SAE; 29.7%) than patients who had a mean RDI of < 85% (AE: 98.0%; SAE: 38.4%) (Table 6).

**Table 6:** Summary of relationship of RDI with treatment outcomes and safety profile (SAS)

	Pazopanib ***N*** = 190		
**Mean RDI (SD)**	79.4 (21.04)		
**Median RDI (range)**	81.9 (32.5–100.0)		
RDI < 85%, n (%)	99 (52.1)		
RDI ≥ 85%, n (%)	91 (47.9)		
Reduced RDI (< 100%), n (%)	120 (63.2)		
**Treatment response categories**	**Mean RDI < 85%** ***n*** **= 73**	**Mean RDI ≥ 85%** ***n*** **= 55**	**Total** ***n*** **= 128**
**ORR, n (%)**	14 (19.2)	18 (32.7)	32 (25.0)
Mean RDI (SD)	63.7 (12.8)	98.2 (3.4)	83.1 (19.4)
**Complete response, n (%)**	1 (1.4)	3 (5.5)	4 (3.1)
Mean RDI (SD)	75.0 (NE)	97.9 (1.8)	92.2 (11.6)
**Partial response, n (%)**	13 (17.8)	15 (27.3)	28 (21.9)
Mean RDI (SD)	62.8 (12.9)	98.2 (3.7)	81.8 (20.1)
**Stable disease, n (%)**	32 (43.8)	24 (43.6)	56 (43.8)
Mean RDI (SD)	61.4 (12.9)	98.1 (3.6)	77.1 (20.8)
**Progressive disease, n (%)**	27 (37.0)	13 (23.6)	40 (31.3)
Mean RDI (SD)	60.8 (14.9)	98.5 (3.2)	73.1 (21.7)
**AE categories**	***n*** **= 99**	***n*** **= 91**	***n =*** **190**
**Any AE, n (%)**	97 (98.0)	70 (76.9)	167 (87.9)
Mean RDI (SD)	61.5 (13.7)	98.2 (3.2)	76.9 (21.1)
**Serious AE, n (%)**	38 (38.4)	27 (29.7)	65 (34.2)
Mean RDI (SD)	61.0 (14.3)	97.8 (3.7)	76.3 (21.4)

*AE* adverse event, *NE* not estimable, *ORR* overall response rate, *RDI* relative dose intensity, *SAS* safety analysis set, *SD* standard deviation

### Long-term response

Of the 109 patients with evaluable data, 39 patients (35.8%) received pazopanib for ≥18 months and 21 remained progression-free at 18 months, with a proportion of 0.19 (19.3%; 95% CI: 12.3–27.9).

### Safety

Overall, 167 patients (87.9%) experienced at least one treatment-emergent AE (TEAE) during the study period, of whom 138 (72.6%) experienced at least one TEAE related to pazopanib. The most common (≥ 15%) TEAEs were diarrhoea (31.1%) and hypertension (15.3%). A total of 65 patients (34.2%) experienced at least one SAE, of whom 13 (6.8%) experienced at least 1 SAE related to pazopanib. Of the 65 patients, 38 (20.0%) died owing to an SAE; three patients (1.6%) died owing to a treatment-related SAE. A total of 36 (18.9%) patients discontinued study treatment owing to a TEAE, of whom 11 (10.9%) discontinued owing to a TEAE related to pazopanib. A total of 77 (40.5%) patients required treatment interruptions or dose adjustments due to a TEAE, of whom 64 (33.7%) required treatment interruptions or dose adjustments due to a TEAE related to pazopanib. TEAEs related to pazopanib are summarised in Table 7.

**Table 7:** Summary of TEAEs related to pazopanib (≥ 5.0%) by maximum toxicity grade (SAS)

Treatment-related TEAE	Pazopanib ***N =*** 190					
	Grade 1 n (%)	Grade 2 n (%)	Grade 3 n (%)	Grade 4 n (%)	Grade 5 n (%)	Overall n (%)
**Number of patients with at least one TEAE**	**103 (54.2)**	**87 (45.8)**	**41 (21.6)**	**0**	**3 (1.6)**	**138 (72.6)**
Hypothyroidism	3 (1.6)	8 (4.2)	0	0	0	11 (5.8)
Diarrhoea	39 (20.5)	12 (6.3)	7 (3.7)	0	0	58 (30.5)
Nausea	15 (7.9)	3 (1.6)	1 (0.5)	0	0	19 (10.0)
Vomiting	10 (5.3)	5 (2.6)	1 (0.5)	0	0	16 (8.4)
Stomatitis	9 (4.7)	2 (1.1)	1 (0.5)	0	0	12 (6.3)
Fatigue	5 (2.6)	10 (5.3)	1 (0.5)	0	0	16 (8.4)
Alanine aminotransferase increased	5 (2.6)	2 (1.1)	4 (2.1)	0	0	11 (5.8)
Aspartate aminotransferase increased	4 (2.1)	5 (2.6)	1 (0.5)	0	0	10 (5.3)
Transaminases increased	4 (2.1)	2 (1.1)	4 (2.1)	0	0	10 (5.3)
Decreased appetite	11 (5.8)	7 (3.7)	0	0	0	18 (9.5)
Palmar-plantar erythrodysesthesia syndrome	21 (11.1)	5 (2.6)	2 (1.1)	0	0	28 (14.7)
Hair colour changes	16 (8.4)	0	0	0	0	16 (8.4)
Rash	8 (4.2)	2 (1.1)	0	0	0	10 (5.3)
Hypertension	3 (1.6)	17 (8.9)	7 (3.7)	0	0	27 (14.2)

*SAS* safety analysis set, *TEAE* treatment-emergent adverse event

### Deaths

A total of 57 patients (30.0%) died during the study. The most common cause of death was disease progression (31 patients [16.3%]), followed by death from unknown causes (8 patients [4.2%]). Three deaths were suspected to be related to pazopanib, including deaths from cerebrovascular stroke, cytomegaloviral pneumonia, and sepsis (*n* = 1 each).

## Discussion

The PARACHUTE study is the largest prospective, observational study of pazopanib aimed to assess the clinical effectiveness of pazopanib in real-world settings in patients with RCC who are naive to VEGFR-TKI therapy in countries in Asia Pacific, North Africa, and Middle East regions, where data from registration trials are lacking or limited. The proportion of patients remaining progression-free at 12 months, i.e., the primary efficacy outcome, was 38.6%, which was within the expected range of 38 to 52% based on the pazopanib pivotal trial [4], but was less than the 45% observed in PRINCIPAL, another prospective observational study of pazopanib [6].

Overall, the effectiveness and safety results from this study are largely consistent with those of the previously reported pazopanib studies [4–6]. A marginally low ORR was observed in this study (24.4% overall and 25.7% for patients with measurable disease) compared with the 30% ORR observed in the PRINCIPAL study [6]. Baseline patient characteristics age < 65 years and favourable risk based on either MSKCC or IMDC appear to be favourable factors to achieve a CR or a PR. The overall DCR observed in this study is the similar to the 68% DCR observed in the pivotal study of pazopanib [4].

The median PFS (9.7 months) in this study is comparable to the PFS noted in the pivotal study and real-world studies reported so far [6, 11, 12]. Baseline patient characteristics associated with PFS benefit included age < 65 years and favourable risk based by either MSKCC or IMDC criteria. Data for risk group classifications were unavailable for about one third of patients (33.7% with unknown MSKCC risk and 38.4% with risk) in this study and are comparable with the percentages reported in In PRINCIPAL study [6] with 27 and 20% and ADONIS study [13] with 46.8 and 48.7% of patients with unknown MSKCC and IMDC risk categories, respectively.

Median PFS for patients with unknown risk group is (months) is comparable with the median PFS of overall population. The proportion of patients remaining progression free for 18 months or longer while on pazopanib in this study was similar to that reported in a long-term response study of sunitinib (19.3% vs 18.9%, respectively) [14].

At the time of start of the PARACHUTE study, pazopanib was considered as one of the standard-of-care first-line therapies for RCC in most countries [7]. The recent development of immune checkpoint inhibitors, either alone or in combination with TKIs, has considerably changed the treatment paradigm of RCC [15–17]. As more treatment options become available, the optimal sequence for metastatic RCC remains unclear. Data on treatment patterns in routine clinical practice provide valuable information to healthcare professionals. In line with the European Society for Medical Oncology (ESMO) clinical practice guidelines for RCC [18], after discontinuation of pazopanib, the largest proportion of patients (40%) switched to nivolumab, an immune-oncology therapy approved for second-line therapy in RCC [19]. The other post-pazopanib treatments included axitinib (25%), everolimus (25%), and sunitinib (17.3%). Fewer than expected (only 9.6%) patients received cabozantinib after progression on pazopanib, with the lack of availability of the drug in many of these countries likely being the cause. Disease progression followed by AEs were the most common reasons for treatment switch.

A considerable proportion of patients (37%) started pazopanib at a reduced dose, with all patients being Asian. One potential explanation for this high percentage could be that most patients (70%) recruited in the study were Asian. Patients receiving a higher mean RDI showed better chances of achieving objective response, indicating a correlation between RDI and treatment outcomes. In contrast to the notion that higher RDI is associated with higher toxicity, patients in the high RDI group reported a better safety profile with fewer AEs and SAEs. Though the performance status was not recorded in this study, it is possible that the physically fit patients might have received higher doses and reported fewer AEs as compared to the lower dose intensity in less fit patients and resulting higher toxicity. The other less likely reason could be that patients on a full dose had a response that might have improved symptoms related to the disease.

With no new or unexpected AEs reported, the safety profile observed in this study is consistent with that seen in the pivotal and real-world evidence studies [15–17]. The most commonly reported AEs related to pazopanib were diarrhoea (30.5%), palmar-plantar erythrodysesthesia syndrome (14.7%), hypertension (14.2%), and nausea (10%). The percentage of patients who discontinued treatment owing to an AE in the current study was approximately 4% higher than that in the PRINCIPAL study. Disease progression was the most common reason for discontinuation as noticed in the pivotal and extension studies of pazopanib [4, 20]. The overall rate of dose changes or treatment interruptions was numerically higher than that observed in the PRINCIPAL study (53.7% vs 49.3%) [6]. Three deaths were suspected to be related to pazopanib, including deaths from cerebrovascular stroke, cytomegaloviral pneumonia, and sepsis.

### Limitations of the study

This study was subject to some limitations, mainly the observational nature and non-interventional design of the study and lack of a control group or tests of hypothesis. However, the non-interventional nature of the study is best suited to obtain real-world data. The representative number of patients observed and the full safety reporting were assets of this study and minimised its limitations. Limitations such as information and selection bias and limitations of feasibility were expected to be similar to those of other multicentre, prospective non-interventional trials.

## Conclusions

The PARACHUTE study represents the real-world evaluation of the effectiveness and safety of pazopanib in patients with advanced or metastatic RCC who are naive to VEGFR-TKI therapy in countries across Asia Pacific, North Africa, and Middle East regions. The results support the use of pazopanib in routine clinical practice and confirm the favourable safety profile of pazopanib consistent with the previously reported safety profile in pivotal and real-world evidence-based studies.

## Data Availability

“Novartis is committed to sharing with qualified external researchers, access to patient-level data and supporting clinical documents from eligible studies. These requests are reviewed and approved by an independent review panel on the basis of scientific merit. All data provided is anonymised to respect the privacy of patients who have participated in the trial in line with applicable laws and regulations. This trial data availability is according to the criteria and process described on www.clinicalstudydatarequest.com.”

## Abbreviations

AE: Adverse event
CI: Confidence interval
CR: Complete response
DCR: Disease control rate
EOS: End of study
ESMO: European Society for Medical Oncology
FAS: Full analysis set
IMDC: International Metastatic Renal Cell Carcinoma Database Consortium
MedDRA: Medical Dictionary for Regulatory Activities
mRCC: Metastatic renal cell carcinoma
MSKCC: Memorial Sloan Kettering Cancer Center
ORR: Objective response rate
PD: Progressive disease
PFS: Progression free survival
PR: Partial response
RDI: Relative dose intensity
RECIST: Response Evaluation Criteria in Solid Tumours
SAE: Serious adverse event
SAS: Safety analysis set
SD: Standard deviation
TEAE: Treatment emergent adverse event
TKI: Tyrosine kinase inhibitor
VEGFR: Vascular endothelial growth factor receptor
