# Correction to: Prospective observational study on Pazopanib in patients treated for advanced or metastatic renal cell carcinoma in countries in Asia Pacific, North Africa, and Middle East regions: PARACHUTE study

**DOI:** 10.1186/s12885-021-08848-8

**Published:** 2021-11-09

**Authors:** Mustafa Erman, Bivas Biswas, Pongwut Danchaivijitr, Lingwu Chen, Yoke Fui Wong, Tarek Hashem, Chun Sen Lim, Bulent Karabulut, Hsiao-Jen Chung, Chandrasekhar Chikatapu, Sara Ingles, Khemaies Slimane, Ravindran Kanesvaran

**Affiliations:** 1 Medical Oncology, Hacettepe University, Ankara, Turkey; 2 Medical Oncology, Tata Medical Center, Kolkata, West Bengal India; 3 Medical Oncology, Siriraj Hospital, Mahidol University, Bangkok, Thailand; 4 Medical Oncology, The First Affiliated Hospital of Sun Yat-sen, Guangzhou, Guangzhou, Guangdong Province China; 5 Radiotherapy and Oncology, National Cancer Institute, Putrajaya, Malaysia; 6 Medical Oncology, Dr. Tarek Hashem’s Clinic, Cairo, Egypt; 7 Clinical Oncology, Sultan Ismail Hospital, Johor Bahru, Malaysia; 8 Medical Oncology, Ege University, Izmir, Turkey; 9 Department of Urology, Taipei Veterans General Hospital and Department of Urology, College of Medicine and Shu-Tien Urological Research Center, National Yang Ming Chiao Tung University, Taipei, Taiwan; 10 Oncology, Novartis Healthcare Pvt. Ltd., Hyderabad, Telangana India; 11 Oncology, Novartis Pharma AG, Basel, Switzerland; 12 Oncology, Novartis Pharma SAS, Rueil-Malmaison, France; 13 Division of Medical Oncology, National Cancer Centre Singapore, Singapore, Singapore

**Correction to: BMC Cancer 21, 1021 (2021)**

**https://doi.org/10.1186/s12885-021-08738-z**

Following publication of the original article [1], the authors identified an error in the author names of Mustafa Erman, Bivas Biswas, Pongwut Danchaivijitr, Lingwu Chen and Chandrasekhar Chikatapu. The given names and family names were erroneously transposed.

Further to this, figure 1 has been updated. The line colors in “C” section for “Poor” and “Unknown” have been changed to align with Figure B and legend to match the line colors.

The author group has been updated above and the original article [1] has been corrected.

**Fig. 1:**
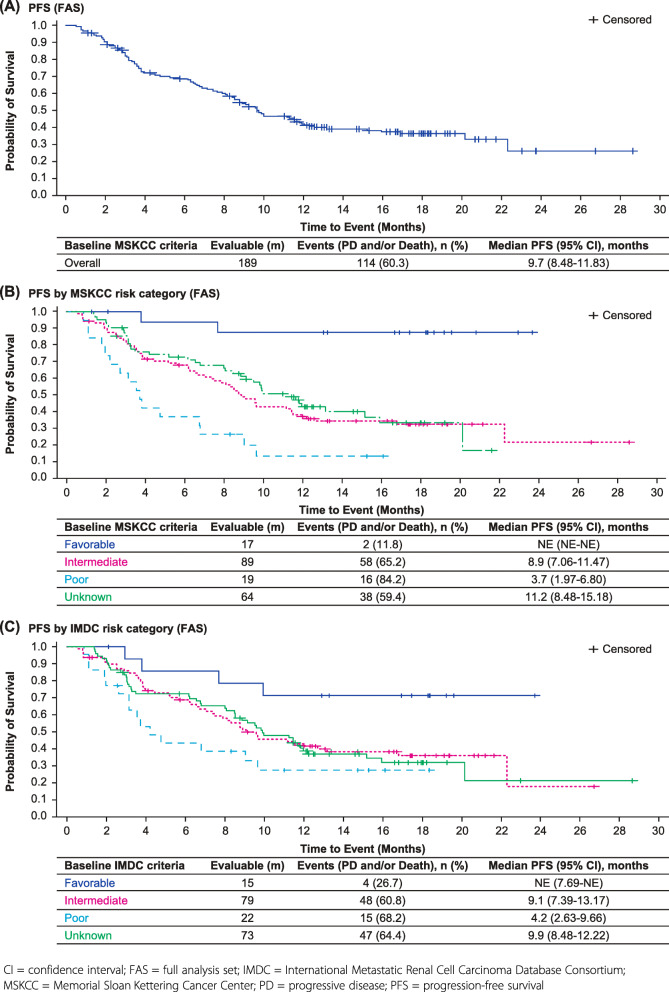
Kaplan-Meier plot for PFS for (A) all patients, (B) MSKCC risk categories, and (C) IMDC risk categories (FAS)
